# Effectiveness of prosthodontic interventions and survival of remaining teeth in adult patients with shortened dental arches—Protocol for a systematic review and meta-analysis

**DOI:** 10.1038/bdjopen.2017.23

**Published:** 2018-04-27

**Authors:** Conor McLister, Michael Donnelly, Christopher Cardwell, Ciaran Moore, Gerald McKenna

**Affiliations:** 1 Centre for Public Health, Queen’s University Belfast, Institute of Clinical Sciences, Belfast, UK

## Abstract

**Aims/Objectives:**

To evaluate studies of the effectiveness of different tooth replacement strategies in adult patients with shortened dental arches (SDA). Specifically, the objectives of the proposed review are to determine the survival rates of different prosthodontic interventions; the risk of tooth loss with and without different prosthodontic interventions; and the impact of different tooth replacement strategies on oral-health related quality of life (OHRQoL).

**Materials and methods:**

The protocol has been registered with the International Prospective Register of Systematic Reviews (PROSPERO), and was developed in accordance with the guidelines of the Preferred Reporting Items for Systematic Review and Meta-analyses Protocols (PRISMA-P). Studies will be selected according to outlined eligibility criteria including types of studies, participants, interventions, comparators and outcomes. Specific search strategies will be created and data collection and analysis will be undertaken by two independent reviewers.

**Discussion:**

The review will assess the body of evidence for clinical decision making in patients with SDA and reduced dentitions, by comparing the effectiveness of different tooth replacement strategies. In addition, it will assess the influence of patients in this decision making, help to inform subsequent cost-effectiveness analyses, identify areas of further research and hopefully inform future healthcare policy.

## Introduction

The population of the world is ageing. The United Nations has estimated that globally, the proportion of older persons (60 years and over) increased from 9.2% in 1990 to 11.7% in 2013. It is expected that this proportion will rise to 21.1% by 2050, with an elderly population of more than 2 billion.^1^ As significant transformations are occurring in populations, changes have also been noted in oral health. More and more adults are retaining their natural teeth into old age. The 2009 UK Adult Dental Health Survey (ADHS) reported that only 6% of those surveyed were missing all their teeth, a significant decrease from 37% in 1968.^2^ With increased tooth retention, population growth and aging, the global burden of oral conditions has increased by ~20.8% since 1990. Collectively, oral conditions affected 3.9 billion people worldwide in 2010.^3^

Potential consequences of tooth loss include impaired mastication, altered food choices, psychosocial problems and reduced oral health-related quality of life.^4,5^ However, depending on the pattern of tooth loss, it may not be necessary to replace missing teeth, especially in older patients. Kayser^6^ first described the shortened dental arch (SDA) concept, suggesting that patients with at least four occlusal units (one unit=pair of occluding premolars; two units=pair of occluding molars) had sufficient adaptive capacity to constitute a functional dentition. The concept has been suggested as an oral health goal for adults until the end of life by the World Health Organisation,^7^ and is considered to have a useful role in contemporary clinical practice.^8^

Where tooth replacement is required to restore partially dentate patients to at least a reduced functional dentition, there are various fixed and removable prosthetic options. Traditionally these have included removable partial dentures, and resin bonded or conventional bridgework. In the last number of decades these options have grown in scope with the demonstrated predictability of dental implants. However, decision making for different patterns of tooth loss and patient groups is often not evidence based.^9^ In addition, the financial cost of tooth loss disproportionately affects older age groups,^10^ and there is a need to achieve better clinical outcomes, which are also cost-effective.

Therefore, the aim of this systematic review is to evaluate studies of the effectiveness of different tooth replacement strategies in adult patients with shortened dental arches. Specifically, the objectives of the proposed review are to determine the:

- survival rates of different prosthodontic interventions;
- risk of tooth loss with and without different prosthodontic interventions; and
- impact of different tooth replacement strategies on oral-health related quality of life (OHRQoL).

## Materials and methods

This protocol has been registered with the International Prospective Register of Systematic Reviews (PROSPERO CRD42017064851), and developed in accordance with the guidelines of the Preferred Reporting Items for Systematic Review and Meta-analyses Protocols (PRISMA-P).^11^

### Eligibility criteria

Studies will be selected according to the criteria outlined below

### Types of studies

We will include studies that used an experimental or observational design:

- randomized controlled trials (RCTs);
- cluster RCTs;
- non-randomized controlled trials (NRCTs);
- controlled before after studies (CBAs); and
- interrupted time series studies (ITSs).

An initial scoping review and the expertise of the review team suggest that it is appropriate to include observational studies as relatively few randomized trials have addressed questions about the effects of interventions.

### Types of participants

Partially dentate male and female adults (18 years or older) will be included. Individuals must have between 4 and 10 functional maxillary and/or mandibular teeth to be eligible.

### Types of interventions

The following prosthodontic interventions will be eligible:

- Acrylic or metal-based removable partial dentures (including those with precision attachments)
- Conventional or resin bonded bridgework (including cantilever and fixed–fixed designs)
- Implant supported crown or bridgework

The comparator will be no intervention or different interventions (‘head-to-head’).

### Types of outcomes

#### Primary outcomes

The primary outcomes of the effectiveness of prosthodontic interventions will be measured in terms of:

- survival of prosthodontic interventions (mean follow-up of 5 years or more);
- survival of remaining teeth (mean follow-up of 5 years or more); and
- change in OHRQoL using validated self-reported measures (mean follow-up of 1 year or more).

#### Secondary outcomes

We will collect other relevant outcome measures including the following:

- Biological complications (dental caries, periodontal/peri-implant disease, loss of tooth vitality, coronal/root fracture)
- Technical complications (loss of retention, fracture/deformation, abutment/screw fracture/loosening)

### Search methods for identification of studies

#### Electronic searches

**Figure 1:**
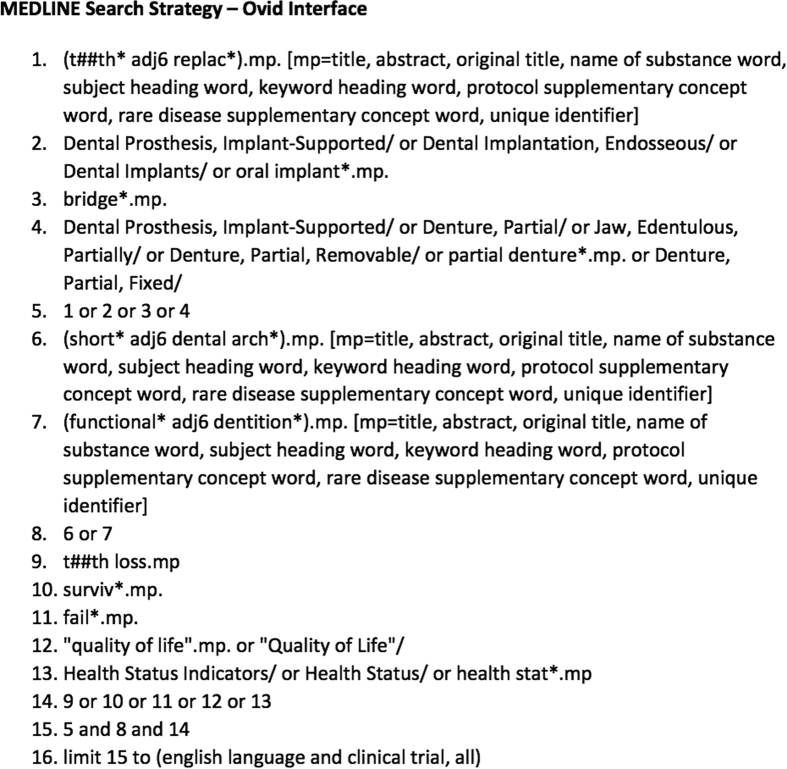
Medline Search Strategy - Ovid Interface

In consultation with the authors, specific search strategies will be created by a health services librarian with expertise in systematic reviewing. A copy of the MEDLINE search strategy is available in Figure 1. We will adapt the MEDLINE strategy for other databases and translate MeSH terms to the controlled vocabularies of those databases as appropriate. We will publish all search strategies used in the review. All databases will be searched from inception to the date of the search.

We will search the following databases for primary studies:

- MEDLINE, 1980 to present, in-process and other non-indexed citations, Ovid SP
- Cochrane Central Register of Controlled Trials (CENTRAL)
- Embase, 1980 to present, Ovid SP

We will search the following trial registries:

- International Clinical Trials Registry Platform (ICTRP), World Health Organization (WHO)
- ClinicalTrials.gov, US National Institutes of Health (NIH)

We will also conduct a grey literature search, using the OpenSIGLE database, to identify studies not indexed in the databases listed above.

#### Searching other resources

To ensure literature saturation, we will review reference lists of included studies or reviews identified through the search. Where necessary, we will also contact the authors of eligible studies and researchers with expertise relevant to the review topic. Authors of relevant studies will be contacted and asked to provide additional data relevant to the review. All strategies used, including a list of sources screened and relevant reviews/primary studies reviewed, will be provided in appendices.

### Data collection and analysis

#### Selection of studies

Two independent reviewers (CML and CM) will screen the titles and abstracts identified by the electronic searches in duplicate. Full reports will be obtained for all titles that appear to meet the inclusion criteria or where there is uncertainty. Additional information from study authors will be requested where necessary. Disagreements between reviewers will be resolved by discussion, and one of two arbitrators (GMK or MD) will adjudicate unresolved disagreements. The reasons for excluding trials will be recorded in the table ‘Characteristics of excluded studies’. We will include, and report characteristics of, studies in the review irrespective of whether measured outcome data are reported in a ‘usable’ way. Studies that meet the inclusion criteria will be included and described in the ‘Characteristics of included studies’ table, even if they do not report usable result. We will document the selection process in sufficient detail to complete a PRISMA flow chart^12^ and a table of ‘Characteristics of excluded studies’.

#### Data extraction and management

We will extract data from included studies and assess their risk of bias. Two review authors (CML and CM) will extract data from each included study independently and in duplicate using a tool developed for the review. We will resolve differences by discussion and, if necessary, arbitration by a third person. For each study with more than one control or comparison group for the intervention, we will extract the results for each intervention arm. We will not double count data within a meta-analysis and we will combine groups to create single pairwise comparisons as appropriate. For each study, we will record the following data:

- Year of publication, country of origin and source of study funding
- Details of the participants, including demographic characteristics and criteria for inclusion
- Details of the study design
- Comparisons and co-interventions
- Details of the outcomes assessed, including method of assessment and adverse outcomes
- Duration of follow-up and assessment of time points

#### Assessment of risk of bias in included studies

Two review authors (CML and CM) will independently assess risk of bias for each randomized controlled study using the criteria outlined in the *Cochrane Handbook for Systematic Reviews of Interventions*.^13^ Domains covered will include:

- sequence generation;
- allocation concealment;
- blinding;
- incomplete outcome data; and
- selective outcome reporting.

We will judge each potential source of bias as ‘unclear’, ‘low risk’ or ‘high risk’, and provide justification in the ‘Risk of bias’ table. The methodological quality of included non-randomized studies will be assessed using the Newcastle-Ottawa scale^14^ for cohort studies. Domains covered will include selection, comparability and outcome.

#### Measures of treatment effect

For dichotomous outcomes (survival of prostheses at time X, tooth survival at time X, biological/technical complications), the odds ratio (and 95% confidence interval) will be extracted from each study, before and after adjustment for any baseline differences (using logistic regression). Time to failure of prostheses or remaining teeth will be compared between intervention and control, by calculating hazard ratios (and 95% confidence intervals). For continuous outcomes (OHRQoL), the difference in mean change (and 95% confidence intervals) will be extracted. These mean differences will be pooled across studies using weighted mean differences or standardized mean differences, if different measurement scales are used. A sensitivity analysis will be included to test the robustness of results by excluding the contribution of studies with an overall high/unclear risk of bias.

#### Unit of analysis issues

All included studies will be assessed to determine the unit of randomization or selection and whether this is consistent with the unit of analysis.

#### Dealing with missing data

To facilitate any meta-analysis, where possible treatment estimates based upon multiple imputation will be used.

#### Assessment of heterogeneity

We will assess the significance of any discrepancies in the estimates of the treatment effects from the different studies by means of Cochrane’s test for heterogeneity (*χ*^2^-test), where *P*<0.1 will be considered significant. We will use the *I*^2^ statistic, which describes the percentage total variation across studies that is due to heterogeneity rather than chance, to quantify heterogeneity, where an *I*^2^ statistic over 50% may represent substantial heterogeneity.^13^

#### Assessment for reporting biases

If there are ⩾10 studies included in any meta-analysis, the potential for publication bias will be explored using funnel plots to evaluate asymmetry.^13^

#### Data synthesis

Expert statistical advice will be sought before undertaking any meta-analyses. If studies are sufficiently homogenous in terms of design and comparator, meta-analyses will be conducted using a fixed or random-effects model. If tests of heterogeneity are not significant, each outcome will be combined and calculated using the fixed effects model. If statistical heterogeneity is observed (*I*^2^>50% or *P*<0.1), the random effects model will be chosen. If heterogeneity is substantial, a meta-analysis will not be performed and a narrative, qualitative summary will be done.

#### Subgroup analysis and investigation of heterogeneity

Subgroup comparisons are by their nature observational and so subject to bias. If there are sufficient studies, we will aim to carry out subgroup analyses to assess clinical heterogeneity, on the following basis:

- Patient characteristics (age, sex, number of teeth)
- Intervention type
- Setting (general dental services, hospital dental services, community dental services)
- Study design

We will only undertake meta-analyses if there are studies of similar comparisons reporting the same outcome measures.

## Discussion

We will use summary tables to present the findings for the main comparisons in the review, to interpret the results and draw conclusions about the effects of different interventions, including the size of the effects and certainty of evidence. The quality of evidence for all outcomes (head-to-head comparisons) will be judged using the GRADE system. Factors considered will include study design, bias, inconsistency and level of precision. Quality will be adjudicated as high (further research is unlikely to change our confidence in the estimate of effect), moderate, low or very low (very uncertain about the estimate of effect). This review will assess the body of evidence for clinical decision making in patients with SDA and reduced dentitions, by comparing the effectiveness of different tooth replacement strategies. Additionally, by including subjective qualitative outcomes, such as OHRQoL, it will assess the influence of patients in this decision making. With evidence of income-related barriers to oral healthcare for many older adults,^10^ the results will help to inform subsequent cost-effectiveness analyses. This review will also help to identify areas of further research and hopefully inform future healthcare policy.
